# Distinct Neuropsychological Mechanisms May Explain Delayed- *Versus* Rapid-Onset Antidepressant Efficacy

**DOI:** 10.1038/npp.2015.59

**Published:** 2015-03-25

**Authors:** Sarah A Stuart, Paul Butler, Marcus R Munafò, David J Nutt, Emma SJ Robinson

**Affiliations:** 1 School of Physiology and Pharmacology, University of Bristol, Bristol, UK; 2 Global Safety Pharmacology, Pfizer Worldwide Research and Development, San Diego, CA, USA; 3 MRC Integrative Epidemiology Unit, UK Centre for Tobacco and Alcohol Studies, and School of Experimental Psychology, University of Bristol, Bristol, UK; 4 Centre for Neuropsychopharmacology, Division of Brain Sciences, Imperial College London, Hammersmith Hospital, London, UK

## Abstract

The biochemical targets for antidepressants are relatively well established, but we lack a clear understanding of how actions at these proteins translate to clinical benefits. This study used a novel rodent assay to investigate how different antidepressant drugs act to modify affective biases that have been implicated in depression. In this bowl-digging task, rats encounter two equal value learning experiences on separate days (one during an affective manipulation and the other during control conditions). This induces an affective bias that is quantified using a preference test in which both digging substrates are presented together and the individual rats’ choices recorded. The assay can be used to measure affective biases associated with learning (when the treatment is given at the time of the experience) or examine the modification of previously acquired biases (when the treatment is administered before the preference test). The rapid-onset antidepressant ketamine, but not the delayed-onset antidepressant, venlafaxine, attenuated the previously acquired FG7142-induced negative bias following systemic administration. Venlafaxine but not ketamine induced a positive bias when administered before learning. We then used local drug infusions and excitotoxic lesions to localize the effects of ketamine to the medial prefrontal cortex and venlafaxine to the amygdala. Using a modified protocol we also showed that positive and negative biases amplified further when the numbers of substrate–reinforcer associations are increased. We propose that this pattern of results could explain the delayed onset of action of venlafaxine and the rapid onset of action but lack of long-term efficacy seen with ketamine.

## INTRODUCTION

Major depressive disorder (MDD) is one of the most significant disorders facing modern society with huge social and economic costs (Moussavi *et al*, 2007; Wittchen *et al*, 2011). Recent developments have revealed impairments in the processing of emotionally relevant information in MDD (Leppänen, 2006; Gotlib and Joormann, 2010; Elliott *et al*, 2011; Roiser *et al*, 2012). These emotional impairments lead to biases in the way that information is processed that influence cognitive processes such as learning and memory, decision making, and attention. For example, patients with MDD are more likely to remember words with a negative valence (Elliott *et al*, 2011; Roiser *et al*, 2012). A cognitive theory of depression was first proposed by Beck in 1967 (Beck, 1967) and biases in many cognitive domains have since been identified in patients with MDD (Leppänen, 2006; Gotlib and Joormann, 2010; Elliott *et al*, 2011; Roiser *et al*, 2012). The potential importance of these neurobiological and emotional processing biases is further highlighted by studies in which effective treatment with antidepressants or deep brain stimulation is associated with remediation of differences in activity in key brain regions such as the subgenual cingulate cortex and amygdala (Ressler and Mayberg, 2007; Hamani *et al*, 2011). These observations suggest that the influence of affective biases on cognitive processes may play an important role in the development and perpetuation of mood disorders (Harmer, 2008; Elliott *et al*, 2011; Roiser *et al*, 2012; Victor *et al*, 2013). Recent human studies have also found that typical antidepressant drugs cause acute changes in these biases that are seen before any subjective improvement in mood (Harmer, 2008; Harmer *et al*, 2009a, b; Victor *et al*, 2013).

**Figure 1:**
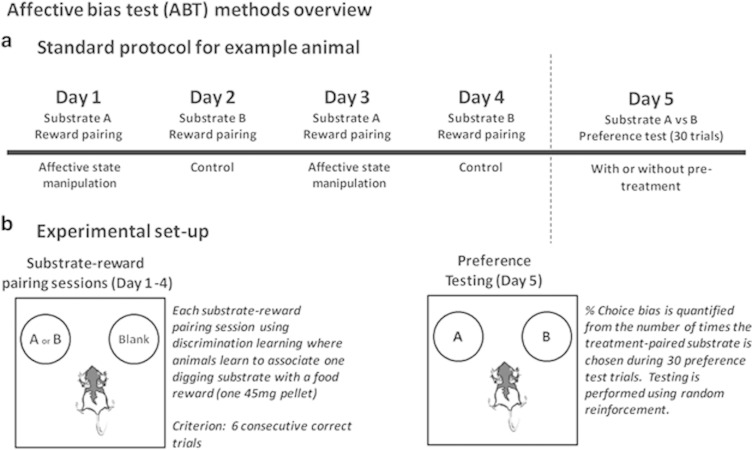
Methods overview. The affective bias test (ABT) provides a translational rodent assay of cognitive affective bias in depression (Stuart *et al*, 2013). An overview of the standard bowl-digging task procedure for a single animal is shown in (a). All studies use a within-subject or mixed study design wherein each animal encounters two independent, learning experiences (substrate–reinforcer pairing sessions, (b), left) over a 4-day period followed by a preference test (b, right). The experiences are of equal value (1 × 45 mg reward pellet) but we have shown that affective states, and antidepressants or prodepressant drugs, induce biases in these experiences that can be quantified using a preference test (Stuart *et al*, 2013).

In this study we have used a translational rodent assay of affective bias (Stuart *et al*, 2013) to investigate whether temporal differences in onset of clinical efficacy seen with delayed-onset (eg, noradrenaline and serotonin reuptake inhibitor venlafaxine) *vs* rapid-onset antidepressant treatments (eg, NMDA antagonist ketamine) (Zarate *et al*, 2006; Mathews and Zarate, 2013) could involve differential modification of these affective biases. The rodent affective bias test (ABT) has shown that experience-dependent learning is biased by affective state or pharmacological treatments, and it exhibits both translational and predictive validity (Stuart *et al*, 2013) (Figure 1a). The ABT uses normal animals and a within-subject design wherein each rat encounters two independent learning experiences (finding food pellet in a specific digging substrate). The two experiences are acquired during discrimination learning sessions under either an affective manipulation or control conditions, and the reinforcer value is kept consistent. Affective bias is quantified in a preference test in which both previously reinforced substrates are presented together and the rat’s choices recorded. A positive affective bias is seen when the rat makes more choices for the experience encountered under treatment *vs* choices for the control substrate, whereas the opposite is seen when the manipulation induces a negative bias (Stuart *et al*, 2013). We first compared the effects of ketamine and venlafaxine in animals in which a negative affective bias had been induced using the benzodiazepine receptor inverse agonist, FG7142 (Stuart *et al*, 2013), or psychosocial stress (Stuart *et al*, 2013). Only ketamine was able to attenuate these negative biases, and hence we next refined its site of action by using local infusions of ketamine into the rat medial prefrontal cortex (including the infralimbic cortex that is homologous to Cg25; Leppänen, 2006; Ressler and Mayberg, 2007). To further elucidate the potential mechanisms involved in ketamine’s effects we also tested two pharmacological manipulations that cause local inactivation: the GABA_A_ agonist muscimol and the local anesthetic bupivicaine. We have previously shown that venlafaxine can induce a positive bias when administered at the time the animal learns the substrate–reinforcer association (Stuart *et al*, 2013); however, ketamine was found to have no effect when administered using the same protocol. In order to further investigate the neural mechanisms involved in the formation of the venlafaxine-induced positive bias, we tested it in animals with or without bilateral excitotoxic lesions of the central nucleus of the amygdala (CeA), a region implicated in learning as well as modulation of stress and arousal systems (Cardinal *et al*, 2002; Balleine and Killcross, 2006). We targeted the amygdala as imaging studies have shown it to be a key region in MDD, emotional processing, and the response to antidepressant treatments (Leppänen, 2006; Ressler and Mayberg, 2007; Victor *et al*, 2010). We also tested these same animals with negative affective state manipulations. In our final set of experiments we investigated whether the magnitude of affective bias is linked to the number of experiences encountered in that affective state. We developed a modified protocol and tested venlafaxine, the cannabinoid_1_ (CB_1_) antagonist/inverse agonist, rimonabant (which is associated with prodepressant effects in humans; Christensen *et al*, 2007; Horder *et al*, 2009), and repeated psychosocial stress based on our previous evidence that these treatments induce positive and negative biases respectively following two substrate–reinforcer experiences (Stuart *et al*, 2013).

## MATERIALS AND METHODS

### Apparatus

The animals were tested in a Perspex arena, 40 cm^2^. The digging substrates (eg, paper bedding, sawdust, sand, cloth, perlite, and so on) were placed in glazed pottery bowls and presented in a pseudorandom order in the left or right position to prevent the rats using spatial cues.

### Subjects

The animals used were 6 cohorts of 16 male Lister-hooded rats weighing ∼300–350 g at the start of dosing (Harlan, UK), housed in pairs under temperature-controlled conditions and a 12 : 12 h light/dark cycle (lights off at 0700 h). They were maintained at ∼90% of their free-feeding weight by restricting access to laboratory chow (Purina, UK) to ∼18 g per rat per day. Water was provided *ad libitum*. All procedures were conducted in accordance with the requirements of the UK Animals (Scientific Procedures) Act 1986 and in accordance with local institutional guidelines. All behavioral testing was carried out between 0900 and 1700 h during the animals’ active phase.

### Affective Bias Test Training and Testing Procedure

The rats were habituated to the test arena and trained to dig in two bowls filled with digging substrate to obtain a quantity of food pellets (45 mg rodent tablet, TestDiet, Sandown Scientific, UK). Training was complete once each rat was able to find the pellets on 12 consecutive trials within 30 s for each trial (full details in Supplementary Materials and Methods). Once trained, each study followed a standard protocol of four pairing sessions followed by a preference test session on the fifth day (Figure 1a). Each pairing session consisted of discrete trials in which the animal was placed into the testing arena and allowed to approach and explore two bowls, one rewarded substrate and the other unrewarded ‘blank’ substrate. Once the animal starting digging in one bowl, the other was removed by the experimenter, the latency to dig recorded, and the trial recorded as correct (rewarded substrate) or incorrect (blank substrate). If the animal failed to approach the bowls and dig within 20 s, the trial was recorded as an omission. Animals were run until they completed six consecutive correct trials. All studies used a within-subject design wherein each animal learnt to associate two different digging substrates (A or B) with a food pellet reward during pairing sessions (see Supplementary Table S1 for the fully counterbalanced study protocol). These pairing sessions were carried out on separate days following either treatment or vehicle. The pairing sessions were carried out on days 1–4 (Figure 1b) and, on day 5, the rats were presented with both reinforced substrates for the first time and their choices over 30 trials recorded (Figure 1b). For the preference test trials, a single pellet was placed in one of the bowls using a random reinforcement protocol such that there was a 1 in 3 probability for each substrate. Trials were run as described above, and the animals’ latency to dig and choice of substrate (A or B) was recorded. In all studies, substrate, pairing session, and treatments (ie, the manipulation used to induce a bias and/or as a pretreatment before recall) were fully counterbalanced (see Supplementary Tables S1–S5 for study design). Results from the preference test day were recorded as number of choices for the vehicle-paired substrate *vs* the number of choices for the treatment-paired substrate, and were used to calculate a %Choice bias value for further analysis.

### Drugs

Drugs for i.p. injection were dissolved in either 0.9% sterile saline (indicated by superscript 1) or a 10% DMSO, 20% cremophor, 80% saline mixture (indicated by superscript 2) in a dose volume of 1ml/kg. Drugs for i.c. infusion were dissolved in 0.1M PBS. Venlafaxine^1^ (3.0 mg/kg, i.p., *t*=−30 min) was purchased from Tocris Bioscience, UK. FG7142^2^ (5.0 mg/kg, i.p., *t*=−30 min), ketamine^1^ (1.0, 3.0 mg/kg i.p., *t*=−60 min, or 1 μg/μl i.c. *t*=−5 min), muscimol (0.1 μg/μl i.c., *t*=−5 min), and bupivacaine (0.75% w/v i.c., *t*=−5 min) were purchased from Sigma Aldrich, UK. Rimonabant^2^ (10.0 mg/kg, i.p*., t*=−30 min) was kindly provided by Pfizer.

### Experiment 1: Effects of Venlafaxine or Ketamine on FG7142 or Psychosocial Stress-Induced Negative Affective Biases

Each study was carried out over 2 weeks using the same cohort of rats and a within-subject study design (Supplementary Table S2). For each of the 2 weeks of the study, a negative affective bias was first induced using either the anxiogenic compound FG7142 (treatment: 5 mg/kg FG7142, i.p., vehicle: DMSO mix) before the pairing sessions or psychosocial stress (treatment: 10 min restraint stress immediately before the pairing session, followed by ~5 h of social isolation, control: normal pair housing). The effects of venlafaxine or ketamine on the negative bias were then tested by administering them before the preference test on day 5. In the first study, animals received either venlafaxine (3 mg/kg, i.p.) or vehicle (saline), counterbalanced over 2 weeks. The dose of venlafaxine was based on our previous experiments (Stuart *et al*, 2013) and had induced a positive affective bias in this assay. In the second study, animals were treated with ketamine (1 mg/kg, i.p.) or vehicle (saline), counterbalanced over 2 weeks. This dose of ketamine was used as it did not cause any sedation as determined by a lack of effect on latency to dig (Supplementary Table S6).

To localize the site and mechanism of action of ketamine, animals were first implanted with mPFC guide cannula (see Supplementary Materials and Methods). The study was carried out over 4 weeks using a within-subject design. A FG7142-induced negative bias was induced each week as described above. Animals were then pretreated with either vehicle (PBS), ketamine (1 μg/μl), bupivicaine (0.75% w/v) or muscimol (0.1 μg/μl) infused 10 min before preference testing on day 5 of each week, using a fully counterbalanced within-subject design (Supplementary Table S3). Drugs were infused through bilateral 33-gauge injectors targeted to the border of the prelimbic and infralimbic cortices. The injector was left in place for 1 min followed by a 2 min infusion (1 μl volume per cannula, 0.5 μl/min) and a further 2 min after infusion. Animals were then returned to their home cage for 5 min before preference testing. Following the completion of the experiment, animals were killed and the tissue fixed and processed for histology (see Supplementary Materials and Methods) and the locations of the final injector tip positions in the mPFC were mapped onto standardized coronal sections of a rat brain stereotaxic atlas.

### Experiment 2: Effects of Venlafaxine and Ketamine on Learning and Induction of an Affective Bias

Previous studies had established a dose-dependent positive bias following administration of venlafaxine before substrate–reinforcer pairing sessions (Stuart *et al*, 2013), but the effects of ketamine had not been tested. The ability or not of ketamine to positively bias new learning was tested using the standard protocol outlined in Figure 1a and described above. Ketamine (0.0, 1.0, 3.0 mg/kg, i.p.) or vehicle (saline) were administered before the substrate–reinforcer pairing sessions using a fully counterbalanced study design (Supplementary Table S4).

To further investigate the neural mechanisms associated with the induction of an affective bias, we tested venlafaxine-induced positive bias and FG7142-induced or psychosocial stress-induced negative bias in animals with lesions to the CeA. As ketamine failed to induce a bias when administered before learning, it was not tested in these animals. Animals received either bilateral excitotoxic lesions (ibotenic acid, 10 μg/μl in 0.1 M PBS, 0.1 μl per site) of the CeA or sham lesions (0.1 M PBS, 0.1 μl per site) (see Supplementary Materials and Methods). Once recovered, the two groups of animals were tested in a series of experiments using the standard ABT protocol. To assess the animals’ ability to develop a bias based on absolute reinforcer value, one substrate was paired with two food pellets and the other substrate with a single food pellet. Preference testing used a single pellet and random reinforcement. For the drug studies, animals from each group underwent pairing sessions in which one substrate (A or B) was paired following pretreatment with either FG7142 (5.0 mg/kg, i.p.), venlafaxine (10.0 mg/kg, i.p.), or vehicle and the other substrate paired with vehicle using a fully counterbalanced study design (Supplementary Table S5). The absolute value of the reinforcer (one pellet) placed in substrates A and B was the same for each session. The same groups of animals were then used in the psychosocial stress study using the restraint stress and social isolation protocol described above. At the end of the study, the tissue was processed for immunohistochemical (NeuN) staining (see Supplementary Materials and Methods).

### Experiment 3: Effects of Repeated Administration of Antidepressant and Prodepressant Manipulations Before Learning

The study investigating the effects of multiple (more than two) pairing sessions after affective manipulation was examined using a modified protocol involving each rat receiving one treatment-pairing session and one vehicle-pairing session each week, followed by a preference test (Figure 4a, full details in Supplementary Table S1). This protocol was repeated each week with the same substrate pairings for 5 consecutive weeks using venlafaxine (3 mg/kg, i.p. *vs* vehicle). The same protocol was also used to test the effects of prodepressant manipulations using once weekly psychosocial stress (*vs* control housing), or the CB1 antagonist, rimonabant (3 mg/kg, i.p., *vs* vehicle). These studies did not include a separate control group but compared experience encountered under manipulation against control/vehicle pairing session (Supplementary Table S1). Previous studies have established the validity of this experiment design (Stuart *et al*, 2013).

### Statistical Analysis

Choice bias was calculated based on the number of choices made for the treatment-paired substrate *vs* the total number of trials (treatment-paired substrate+vehicle-paired substrate). A value of 50 was then subtracted from the choice bias score to give a %Choice bias where a bias toward the treatment-paired substrate gave a positive value and a bias toward the control-paired substrate gave a negative score. Latency and trials to criterion were recorded during pairing sessions in the ketamine dose–response study and the CeA lesion studies and analyzed to determine whether there were any nonspecific effects of treatment (eg, sedation, anorexia). Statistical analyses were performed using Graphpad Prism version 6. The %Choice bias data from experiment 1 were analyzed using paired *t*-tests. Experiments 2 and 3 were analyzed using a repeated measures ANOVA with TREATMENT as factor. The %Choice bias data from experiment 4 used a one-way ANOVA with TREATMENT as the within-subjects factor and GROUP as a between-subjects factor. The *post hoc* analysis for each treatment used a one-sample *t*-test against a theoretical mean of 0% choice bias where 0% is equivalent to 15 choices for the treatment-paired substrate and 15 choices for the vehicle-paired substrate. Between-treatment comparisons were made using a paired or unpaired *t*-test as appropriate. Analysis of the choice latency and trials to criterion was made using a paired *t*-test comparing drug with vehicle for the pairing sessions.

## RESULTS

### Experiment 1: Effects of Venlafaxine or Ketamine on FG7142 or Psychosocial Stress-Induced Negative Affective Biases

**Figure 2:**
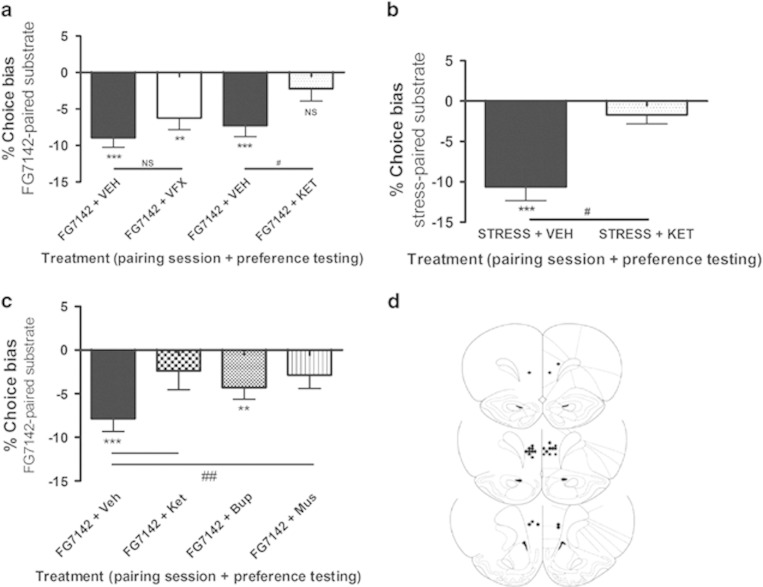
Ketamine but not venlafaxine attenuates negative cognitive affective biases. FG-7142 treatment (5 mg/kg) administered before one of the substrate–reinforcer pairing sessions induced a negative affective bias during preference testing that was attenuated by systemic administration of ketamine (1 mg/kg) but not venlafaxine (3 mg/kg) (a). Systemic administration of ketamine also attenuates psychosocial stress-induced negative affective bias when administered before preference testing (b). The effects of systemic treatment with ketamine were replicated when the drug was targeted specifically to the medial prefrontal cortex (mPFC) (c). In vehicle-infused animals, FG7142 induced a negative affective bias consistent with previous studies. There was a main effect of TREATMENT, and *post hoc* pairwise comparison with vehicle-infused animals revealed that FG7142-induced negative affective bias was attenuated when animals received mPFC infusions of ketamine (1 μg/μl) or muscimol (0.1 μg/μl) (c). The location of the injector placement was confirmed post-mortem and black dots represent the location of the cannula tip as assessed from Cresyl violet-stained brain sections (d). Coronal sections are +4.2 mm to +3.2 mm relative to bregma (Paxinos and Watson, 1998). Data shown as mean±SEM, *n*=14–16 (two animals were excluded from the infusion study because of incorrect placement of the injector). **p<0.01, ***p<0.001 *vs*. theoretical mean of 0% choice bias, ^#^p<0.05, ^##^*p*<0.01 *vs* vehicle (systemic or mPFC infusion, paired *t*-test), BUP, bupivicaine; KET, ketamine; MUS, muscimol; VEH, vehicle.

The FG7142-induced negative bias was attenuated when animals received systemic ketamine before preference testing (paired *t*-test: *t*_15_=2.3, *p*=0.038 *vs* vehicle, *n*=16; Figure 2a). In contrast, no attenuation was observed with venlafaxine pretreatment (*t*_15_=1.71, *p*=0.109 *vs* vehicle, *n*=16, Figure 2a). Following administration of ketamine but not venlafaxine, animals exhibited neutral responding suggesting that the FG7142-induced negative bias was prevented by this rapid-onset antidepressant (one-sample *t*-test: FG7142+vehicle (*t*_15_=4.9, *p*=0.0002 and *t*_15_=7.0, *p*<0.001); FG7142+venlafaxine (*t*_15_=4.0, *p*=0.0013); FG7142+ketamine (*t*_15_=1.5, *p*=0.16), *n*=16 per group; Figure 2a). As we have shown previously (Stuart *et al*, 2013), psychosocial stress also induced a significant negative affective bias when the animals received vehicle treatment before recall (one-sample *t*-test: *t*_15_=6.2, *p*<0.0001, *n*=16; Figure 2b). This effect was attenuated by ketamine administration before the preference test (paired *t*-test: *t*_15_=6.6, *p*<0.0001, *n*=16; Figure 2b), suggesting that the ability of ketamine to attenuate negative affective biases is observed for both pharmacological- and nonpharmacological-induced negative biases.

### Effects of Medial Prefrontal Cortex Infusions and Negative Biases

There was a main effect of treatment during preference testing (RM ANOVA: F_3, 39_=2.9, *p*=0.048, *n*=14, Figure 2c) with FG7142 inducing a negative bias in animals receiving a vehicle infusion before preference testing (*post hoc* one-sample *t*-test: *t*_13_=5.4, *p*=0.0001, *n*=14). Both ketamine and muscimol infusions attenuated the FG7142-induced negative bias when compared with vehicle infusions (paired *t*-test: ketamine (*t*_13_=3.3, *p*=0.0058, *n*=14), muscimol (*t*_13_=3.1, *p*=0.009, *n*=14); Figure 2c). Moreover, neither treatment was associated with a negative bias, with infusions of ketamine or muscimol effectively leading to neutral responding (*post hoc* one-sample *t*-test: ketamine (*t*_13_=1.1, *p*=0.29), muscimol (*t*_13_=1.83, *p*=0.09), *n*=14; Figure 2c). Bupivicaine also appeared to attenuate the FG7142-induced negative bias, although pairwise comparisons between the vehicle- and bupivicaine-infused groups provided only weak statistical evidence for this (paired *t*-test: *t*_13_=1.8, *p*=0.092, *n*=14). Thus, both ketamine infusions into the prefrontal cortex and pharmacological inactivation of this region prevented negative biases associated with previous experiences. Location of the final injector position is shown in Figure 2d.

### Experiment 2: Effects of Venlafaxine and Ketamine on Learning and Induction of an Affective Bias

**Figure 3:**
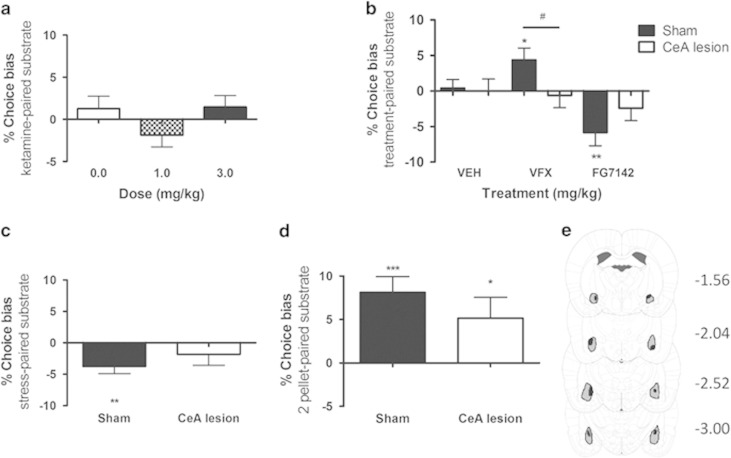
Lesions of the amygdala prevent the development of cognitive affective biases involving new learning. In contrast to the effects previously observed for venlafaxine (Stuart *et al*, 2013), systemic ketamine (1.0 and 3.0 mg/kg) treatment before the substrate–reinforcer pairing sessions failed to have any effect on new learning as shown by the lack of any bias during preference testing (a). In animals with excitotoxic lesions targeted to the CeA, the venlafaxine-induced positive affective bias was attenuated. The *post hoc* pairwise comparisons revealed that venlafaxine’s effects were completely prevented in the lesioned animals. Pairwise comparisons between sham and lesion groups following the negative affective manipulations were not different, although in the lesion group, a negative bias was not observed (FG7142 (b), restraint stress and social isolation (c)). Interestingly, lesions to the CeA do not affect choice bias associated with absolute changes in reinforcer outcome during learning (d). Schematic representation of excitotoxic lesions to the CeA (e). Shaded areas represent the smallest (black) and largest (gray) extent of neuronal damage quantified from post-mortem neuN-stained sections. Coronal sections are −1.56 mm to −3.00 mm relative to bregma (Paxinos and Watson, 1998). Data shown as mean±SEM, sham: *n*=16, lesion *n*=11 (5 animals were excluded because of unilateral or lesions extending beyond CeA). *P<0.05, **p<0.01, ***p<0.001 *vs* theoretical mean of 0% choice bias, ^#^*p*<0.05 unpaired *t*-test. CeA, central nucleus of the amygdala; VEH, vehicle; VFX, venlafaxine.

Acute treatment with ketamine before learning did not induce an affective bias during preference testing (RM ANOVA: F_2, 30_=1.97, *p*=0.16, *n*=16; Figure 3a). The higher dose of ketamine (3 mg/kg) increased response latency during the pairing sessions (Supplementary Table S6).

### Effects of Lesions to the CeA on the Induction of Positive and Negative Affective Biases

Analysis of histological rat brain sections showed that a total of 11 out of 16 animals sustained bilateral lesions to the CeA. The extent of the lesions included in the analysis is illustrated in Figure 3e.

There was a main effect of drug treatment (F_2, 50_=7.5, *p*=0.001, sham: *n*=16, lesion: *n*=11) and drug × lesion interaction (F_2, 50_=4.7, *p*=0.014, sham: *n*=16, lesion: *n*=11), although there was no main effect of lesion (F_1, 50_=0.18, *p*=0.69, sham: *n*=16, lesion: *n*=11). Treatment with venlafaxine induced a positive affective bias in the sham group consistent with our previous studies (Stuart *et al*, 2013); however, these effects were attenuated in the group with amygdala lesions (unpaired *t*-test: *t*_25_=2.4, *p*=0.023, sham: *n*=16, lesion: *n*=11; Figure 3b). Using either FG7142 (Figure 3b) or restraint stress and social isolation (Figure 3c), we also found that animals in the lesion group did not develop negative biases to these manipulations (one-sample *t*-test: FG7142 (*t*_10_=1.4, *p*=0.20), stress (*t*_10_=1.0, *p*=0.33), *n*=11), although the effects were more variable and there was no evidence that they were different in pairwise comparisons with sham animals (unpaired *t*-test sham *vs* lesion: FG7142 (*t*_25_=1.27, *p*=0.21), stress (*t*_25_=0.97, *p*=0.34), sham: *n*=16, lesion: *n*=11). Both manipulations did induce a negative bias in the sham animals (one-sample *t*-test: FG7142 (*t*_15_=3.1, *p*=0.0069), stress (*t*_15_=3.3, *p*=0.0048), *n*=16). Lesioning the amygdala did not affect choice latency or trials to criterion (Supplementary Table S7) or the animal’s ability to form a bias based on an absolute difference in reinforcer value (one-sample *t*-test: sham (*t*_15_=4.5, *p*=0.0005, *n*=16), lesion (*t*_10_=2.2, *p*=0.049, *n*=11); unpaired *t*-test sham *vs* lesion: *t*_25_=0.90, *p*=0.38; Figure 3d).

### Experiment 3: Effects of Repeated Administration of Antidepressant or Prodepressant Manipulations before Learning

**Figure 4:**
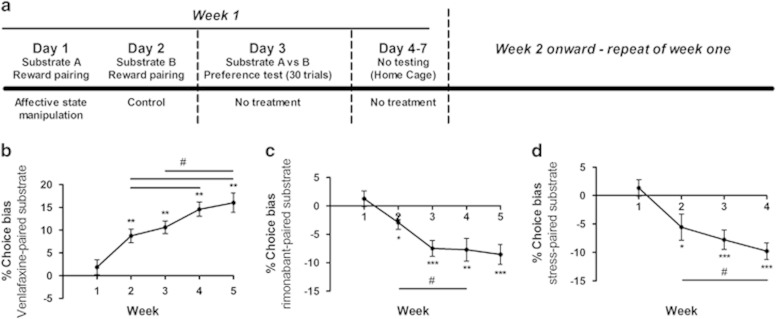
Affective biases amplify with additional learning experiences. Utilizing a modified protocol (a), the effects of additional substrate–reinforcer pairing sessions were investigated. The effects of venlafaxine (b) treatment further amplified when the number of substrate–reinforcer pairing sessions was increased. The effects were significant from week 2 onward, consistent with the standard protocol (Figure 1a), and this increased further with each successive postdrug encounter with the reinforcer-paired substrate. Using the same procedure the prodepressant drug treatment rimonabant (c) and psychosocial stress manipulation (d) were shown to induce a negative bias that also amplified with each successive experience. Data shown as mean±SEM, *n*=15–16. Data analyzed using repeated measures ANOVA, *p<0.05, **p<0.01, ***p<0.001 *vs* theoretical mean of 0% choice bias, ^#^*p*<0.05 paired *t*-test.

The results in Figure 4b show how positive affective bias during venlafaxine treatment increases with each successive treatment (RM ANOVA: F_4, 60_=25.6, *p*<0.0001, *n*=16). Venlafaxine treatment induced a positive choice bias after week 2 (equivalent to the ketamine study protocol), and this bias continued to increase with each successive postdrug experience over 5 weeks of study (one-sample *t*-test: week 1 (*t*_15_=1.16, *p*=0.26), week 2 (*t*_15_=5.77, *p*<0.0001), week 3 (*t*_15_=7.64, *p*<0.0001), week 4 (*t*_15_=9.25, *p*<0.0001), week 5 (*t*_15_=16.04, *p*<0.0001)).

Opposite to the effects observed with venlafaxine treatment, repeated psychosocial stress induced a negative bias that increased with each successive treatment (F_3, 42_=11.2, *p*<0.0001, *n*=16; Figure 4d). A similar effect was seen when animals received repeated treatments with the CB_1_ receptor antagonist/inverse agonist, rimonabant (F_4, 60_=10.2, *p*<0.0001, *n*=16; Figure 4c). For both treatments, a negative affective bias was observed after two exposures and further increased in magnitude with each successive treatment (one-sample *t*-test: stress week 1 (*t*_15_=0.92, *p*=0.37), week 2 (*t*_15_=2.42, *p*=0.029), week 3 (*t*_15_=4.55, *p*=0.0005), week 4 (*t*_15_=6.64, *p*<0.0001); rimonabant week 1 (*t*_15_=0.92, *p*=0.37), week 2 (*t*_15_=2.41, *p*=0.03), week 3 (*t*_15_=5.32, *p*<0.0001), week 4 (*t*_15_=3.88, *p*=0.0015), week 5 (*t*_15_=4.89, *p*=0.0002), *n*=16).

## DISCUSSION

These studies provide a systematic series of animal experiments investigating neuropsychological mechanisms that have been linked to depression and antidepressant drug efficacy. Overall, these findings suggest that delayed- and rapid-onset antidepressant treatments modify affective biases through distinct mechanisms involving different brain regions (Table 1). Specifically, the rapid-onset antidepressant ketamine attenuated previously acquired negative biases through effects in the medial prefrontal cortex but failed to induce a bias when administered before learning. In contrast, the delayed-onset antidepressant venlafaxine induced a positive bias when administered before learning through effects in the amygdala but failed to attenuate previously learnt negative biases. Taken together, these findings suggest that the ability of drugs to modify either new learning or previously acquired affective biases may contribute to the temporal differences in their efficacy in depression.

### Neuropsychological Effects of Ketamine and Rate of Onset of Action in MDD

The NMDA receptor antagonist ketamine induces an antidepressant effect in patients within a few hours of treatment with effects lasting for up to a week (Zarate *et al*, 2006). Previous studies have shown that these effects are linked to synaptic mechanisms in the medial prefrontal cortex involving the mTOR pathway (Li *et al*, 2010), although how these biological changes relate to the emotional symptoms of depression has not been elucidated. In contrast, drugs such as venlafaxine have a delayed onset of action with clinical benefit taking several weeks of treatment. The results from the ABT suggest that the neuropsychological effects of ketamine could be mediated by disruption to neurotransmission in the medial prefrontal cortex leading to a remediation of negative biases. The effects of ketamine in the ABT were not specific to an NMDA-mediated mechanism and a similar result was seen when animals received an infusion of the GABA_A_ agonist, muscimol to induce a temporary pharmacological lesion. At low doses, ketamine is known to increase cortical glutamate (Stone *et al*, 2012) through disinhibition of GABA interneurons (Moghaddam *et al*, 1997; Homayoun and Moghaddam, 2007), an effect that may lead to disruption in neurotransmission in regions including the subgenual cingulate where altering activity has been linked to antidepressant efficacy with drug treatments (Ressler and Mayberg, 2007; Hamani *et al*, 2011) or deep brain stimulation (Ressler and Mayberg, 2007). The effects of bupivacaine were inconclusive, although they suggest that blocking transmission in this region, through effects on both cell bodies and fibers of passage, has a similar effect on negative biases. Given that deep brain stimulation has been shown to have a more rapid onset of action as well as efficacy in treatment-resistant populations, our results support a mechanism involving a disruption of transmission in this region that results in an attenuation of negative processing biases. As negative affective bias is a prevalent feature in depression (Leppänen, 2006; Gotlib and Joormann, 2010; Elliott *et al*, 2011; Roiser *et al*, 2012), these findings suggest that attenuation of negative bias may represent a neuropsychological mechanism through which ketamine exerts its rapid antidepressant effects. Although the effects of ketamine have been seen as an antidepressant effect in patients, the results observed in these animal studies suggest that the main effect is to neutralize negative biases. This is a similar idea to that proposed in a previous clinical study with ketamine (Abel *et al*, 2003). Our studies cannot exclude other mechanisms, including a generalized effect on memory, although studies using similar doses in rats (Ribeiro *et al*, 2013) and humans have not found that ketamine at these low doses has a specific amnesic effect (Morgan *et al*, 2004).

In contrast to the results observed using venlafaxine, ketamine treatment lacked the ability to modify learning associated with new experiences. The long-term efficacy of ketamine treatment may therefore be limited not only by its propensity to induce psychosis but also because it lacks the ability to modify learning in a positive direction. These findings highlight a potential limitation associated with drugs such as ketamine, as a lack of long-term efficacy is predicted given their inability to affect new learning. However, the combination of both an ability to block previously acquired negative biases with the ability to positively bias learning associated with new experiences may provide a rapid onset of action with long-term efficacy in treating depression. The challenge will be to find a drug, or combinations of drugs, that can achieve this.

### Antidepressant and Prodepressant Drug Treatments and Experience-Dependent Learning

In contrast to the effects seen with ketamine, the serotonin and noradrenaline reuptake inhibitor venlafaxine failed to attenuate the negative bias induced by FG7142 treatment when administered before the preference test. However, previous studies using the ABT have shown that a wide range of typical and atypical antidepressant treatments positively bias experience-dependent learning (Stuart *et al*, 2013). These animal studies build on existing clinical data and show that, as well as modulating the processing of emotional information (Harmer, 2008; Harmer *et al*, 2009a; Pringle *et al*, 2011), antidepressants like venlafaxine also affect experience-dependent learning and memory. The effect of treatment appears to be to enhance the relative value attributed to that experience and increase the likelihood of the animal choosing the associated cue when compared with a cue paired under neutral conditions. In this study, the effects of venlafaxine were further investigated using an additive study in which the treatment-paired experience was repeated each week but with a 7-day period between drug administrations. The results showed that each time the experience was encountered under drug treatment, the subsequent positive bias observed increased. As seen in our previous studies, two experiences of the substrate–reinforcer association were required before a positive bias was seen but this then amplified with successive pairing sessions. In terms of the symptoms of depression, these effects may be very important, as enhancing the value associated with an experience will influence subsequent motivations to repeat the activity that led to it. These effects of venlafaxine may counteract the negative biases observed in depressed patients that, through new learning, gradually increase the patient’s ability to experience reward and motivation to engage in rewarding activities. However, our results suggest that the lack of ability of venlafaxine to attenuate previously acquired negative affective biases may contribute to its delayed onset of action.

Although repeated venlafaxine treatment led to an increase in the magnitude of positive bias observed, the same study design carried out using prodepressant manipulations had the opposite effect. Consistent with our previous data using prodepressant manipulations, both rimonabant and exposure to psychosocial stress were associated with inducing a negative cognitive affective bias. In this study, the treatments used the same extended protocol as the venlafaxine study and revealed a similar amplification of the effect observed with each successive pairing session. Although these experiments are in normal animals, they suggest that experiences encountered during these manipulations are negatively biased and this bias amplifies with each treatment. As both stress (Kessler, 1997) and rimonabant treatment (Rumsfeld and Nallamothu, 2008) are linked to an increased risk of developing depression, our findings support the hypothesis that negative cognitive affective biases may contribute to the development of mood disorders.

### The Role of the CeA in the Formation of Cognitive Affective Biases

The finding that bilateral lesions of the amygdala impair the ability of venlafaxine to induce a positive affective bias implicates a key role for this region in mediating the positive affective biases associated with antidepressant treatment and learning and memory. Imaging studies in MDD have previously linked amygdala dysfunction with the disease and emotional processing biases (Hamilton *et al*, 2008; Victor *et al*, 2010). The results from the ABT suggest that the ability of venlafaxine to positively bias experience-dependent learning and memory requires an intact CeA. The CeA has been previously linked to stress and depression (Herman and Cullinan, 1997; Roozendaal *et al*, 1997). The present findings suggest that the link between the neurochemical effects of antidepressant drug treatments and the treatment of MDD may involve positive biases mediated at least in part by the CeA. The results for the negative state manipulations in the lesioned animals were inconclusive. Although FG7142 and psychosocial stress did not induce negative biases in the lesioned animals, there was no overall difference when compared directly with the sham animals. At this stage it is not clear whether negative biases involve additional brain regions or whether the assay is limited in terms of its sensitivity when used in these more complex study designs. Interestingly, the control experiment wherein the absolute value of the reinforcer was modified revealed that both lesioned and sham animals were able to develop a positive bias. Absolute reinforcer information processing has been more commonly associated with the basolateral nucleus of the amygdala (Blundell *et al*, 2001) that was not shown to be affected in the post-mortem analysis. These findings suggest that different distinct neural mechanisms are involved in the antidepressant-induced positive biases associated with experience-dependent learning observed in this assay *vs* those arising from differences in absolute reinforcer value.

### Summary

Together, our findings suggest that the modulation of affective biases by a drug may be an important neuropsychological mechanism in achieving efficacy in depression as well as influencing the rate of onset of clinical benefit. Our observations correspond well with patient and healthy volunteer data that show that delayed-onset and rapid-onset antidepressants have acute effects on neuropsychological processes (Harmer *et al*, 2009a; Victor *et al*, 2013). Using animals, we are able to reveal how delayed- *vs* rapid-onset antidepressants differentially modulate affective biases, and these effects could explain the differences in their rate of onset and long-term efficacy. Although these studies are limited by the fact that they are carried out in normal animals and only model the symptom of affective biases associated with learning and memory, our work adds to a growing literature that suggests that neuropsychological mechanisms are important to both the development of depression and its treatment with antidepressant drugs. Further studies are also needed to understand the neural circuits involved and whether the mechanisms that underpin the formation of cognitive affective biases are dissociable from those that are related to their influence of subsequent behaviors.

## Funding and Disclosure

SAS is funded by a BBSRC Industrial CASE studentship with Pfizer, UK. This work was also funded by an RCUK academic fellowship awarded to ESJR with additional financial support provided by the British Pharmacological Society Integrative Pharmacology Fund and the Wellcome Trust (reference no. 084621/Z/08/Z). MRM is a member of the United Kingdom Centre for Tobacco and Alcohol Studies, a UKCRC Public Health Research: Centre of Excellence. Funding from the British Heart Foundation, Cancer Research UK, Economic and Social Research Council, Medical Research Council, and the National Institute for Health Research, under the auspices of the UK Clinical Research Collaboration, is gratefully acknowledged. DJN is an advisor to British National Formulary, MRC, GMC, Department of Health, President of European Brain Council, Past President of British Neuroscience Association and European College of Neuropsychopharmacology, Chair of the Independent Scientific Committee on Drugs (UK), Member of International Centre for Science in Drug Policy, Advisor to Swedish government on drug, alcohol, and tobacco research, editor of the *Journal of Psychopharmacology*, Member of Advisory Boards of Lundbeck, MSD, Nalpharm, Orexigen, Shire, MSD, has received speaking honoraria (in addition to above) from BMS/Otsuka, GSK, Lilly, Janssen, Servier, AZ, and Pfizer, is a member of the Lundbeck International Neuroscience Foundation, has received grants or clinical trial payments from P1vital, MRC, NHS, Lundbeck, RB, has share options in P1vital, has been an expert witness in a number of legal cases relating to psychotropic drugs, and has edited/written 27 books, some purchased by pharma companies. PB is a current employee of Pfizer.

## Figures and Tables

**Table 1:** Summary of Key Findings

	**Effect on FG7142-induced negative bias**	**Effect on stress-induced negative bias**	**Expression of affective bias**
Venlafaxine	No effect	N/D	Positive bias^a^
Ketamine	Attenuation^b^	Attenuation	No effect
Rimonabant	N/D	N/D	Negative bias
Psychosocial stress	N/D	N/D	Negative bias

a Mechanism involving the central nucleus of the amygdala.

b Mechanism involving the medial prefrontal cortex.
