# Supplementary material for: Distinct Neuropsychological Mechanisms May Explain Delayed- Versus Rapid-Onset Antidepressant Efficacy
Source: Neuropsychopharmacology. 2015 Mar 25;40(9):2165–74. doi: 10.1038/npp.2015.59 (PMC4487826; doi:10.1038/npp.2015.59)
Supplement: Supplementary Information [file npp201559x1.docx]

**SUPPLEMENTARY MATERIALS AND METHODS**

**Affective bias test (ABT) training and testing procedure**

The animals were trained and tested in a Perspex arena, 40 cm^2^ (Fig 1B). The substrates e.g. bedding, sawdust, sand, cloth, perlite etc, were placed in glazed pottery bowls (5 inch) and presented in a pseudo-random spatial order to prevent rats using spatial cues to select the correct substrate.

**Training:** The rats were habituated to the test arena and trained to dig in two bowls filled with sawdust to obtain a quantity of food pellets (45mg rodent tablet, TestDiet). Each stage of training is described in the table below.

| **Day 1: Arena habituation** | Exploration of two bowls each containing 3 food pellets.  The rat is allowed to approach both bowls and eat all the pellets.  When all pellets are eaten the rat is removed, the bowls re-baited and the rat placed back into the arena. Criteria: total of 10 mins exploration. |
| --- | --- |
| **Day 2: Digging training 1** | Exploration of two bowls each containing 1cm sawdust and 3 food pellets (1 on surface and 2 buried).  Each rat is allowed to dig in both bowls to find the pellets.  When all pellets are eaten the rat is removed, the bowls re-baited and the rat placed back into the arena. Criteria: completion of 12 discrete trials (max. 2 min each trial) |
| **Day 3: Digging training 2** | Choice of two bowls each containing 2cm sawdust and 2 buried food pellets.  Each rat is allowed to dig in one bowl to find the pellets. The other bowl is removed when the rat makes a choice (ie. Digs in a bowl).  Criteria: completion of 12 discrete trials (max. 30 sec each trial). |
| **Day 4: Digging training 3** | Choice of two bowls each containing 2cm sawdust and 2 buried food pellets.  Each rat is allowed to dig in one bowl to find the pellets. The other bowl is removed when the rat makes a choice (ie. Digs in a bowl).  Criteria: completion of 12 discrete trials (max. 30 sec each trial). |
| **Day 5: Novel substrate** | Exploration of two bowls containing a novel digging substrate (eg. Mouse bedding) and 2 buried food pellets  Each rat is allowed to dig in one bowl to find the pellets. The other bowl is removed when the rat makes a choice  Criteria: completion of 12 discrete trials (max. 30 sec each trial). |

**Table 1: Summary of bowl-digging training for the ABT.**

**General Protocol:** Each study followed a standard protocol of four pairing sessions followed by a preference test session on the fifth day (Fig 1A). All drug studies were carried out blind to treatment. Animals were randomly assigned to one of four counterbalanced groups to prevent bias associated with substrate, drug or treatment day. Each pairing session consisted of individual trials in which the rat was required to choose between one of the two bowls to locate a food pellet reward (Fig 1B). In each of these trials, one of the bowls contained a ‘reward-paired’ substrate and the other contained a different, ‘blank’ substrate. The blank substrate was the same for all four pairing sessions. In the blank substrate, the equivalent number of food pellets was crushed into the bowl to avoid discrimination from the reward-paired substrate based on odour. The rat was placed in front of the two bowls and allowed to dig in one of the two bowls. Once the animal began to dig, the other bowl was removed from the test arena. Digging in the reward-paired substrate was recorded as a correct trial, and digging in the blank substrate was recorded as an incorrect trial. The latency to dig was also recorded for each trial and the session was completed once the rat reached a criterion of 6 consecutive correct trials (probability of by chance being 0.015). The second pairing session followed the same protocol, but the rats were presented with the second reward-paired substrate. The pairing sessions were repeated to give a total of four sessions on consecutive days. On the fifth day, the rats were presented with both reward-paired substrates for a total of 30 trials (Fig 1B). A single pellet reward was placed in one of the bowls using a random reinforcement protocol such that there was a 1 in 3 reward probability for each substrate. The random reinforcement schedule was used to maintain animals responding during the preference test but reduce the potential confounds associated with new learning.

**Experiment 1: Effects of venlafaxine or ketamine on FG7142 or psychosocial stress-induced negative affective biases.**

**mPFC cannulation:** Rats were anesthetised with isoflurane/O2 and secured in a stereotaxic frame. Bilateral 32-gauge guide cannulae (Plastics One, UK) were implanted in the mPFC according to the stereotaxic coordinates: anteroposterior +3.0mm, lateral ±0.75mm and dorsoventral −2.0mm from bregma (Paxinos and Watson, 1998). The cannulae were secured to the skull with dental acrylic and stainless steel screws. After surgery, the animals were housed individually overnight then allowed 4–7 days recovery in normal paired housing conditions. Following post mortem histology, two animals were excluded from the analysis due to incorrect cannula placement.

#### Infusion Procedure: Following the recovery period rats were habituated to the infusion procedure during two sessions where animals were lightly restrained and the cannula dummy removed and then replaced. In a third habituation session animals received a vehicle (phosphate-buffered saline, PBS) infusion. During experimental infusions, the rats were gently restrained while the cannula dummy was removed and a 33-gauge bilateral injector extending 2.5mm beyond the length of the guide cannula was inserted into the mPFC. The injector was left in place for 1 min prior to infusions of vehicle or drug (1.0μl) over 2 min. The injector was left in place for a further 2min to allow diffusion of the drug into the tissue surrounding the injector, and then the injector was removed and the dummy replaced.

#### Experimental Summary: Reward-pairing sessions were as described previously except one substrate (A or B) was paired following pre-treatment with FG7142 (5.0mg/kg) versus vehicle treatment. The absolute value of the reward (1 pellet) placed in substrates A and B was the same for each session. Prior to the preference test on day 5, animals were given infusions of either vehicle (phosphate-buffered solution), ketamine (1μg/ul), bupivicaine (0.75%) or muscimol (0.1μg/ul) then returned to their homecage for 5min before testing.

#### Histology: Following the completion of the experiment, the animals were anesthetised with a lethal dose of sodium pentobarbitone (0.5ml Euthatal, 200mg/ml, Genus Express, UK) and perfused via the left ventricle with 0.01M PBS followed by 4% paraformaldehyde. The brains were removed and postfixed in paraformaldehyde. Prior to being cut, the brains were transferred to 30% sucrose in 0.1M PBS and left for 2 days. Coronal sections were cut at 40μm on a freezing microtome and stained with Cresyl Violet. Locations of the final injector tip positions in the mPFC were mapped onto standardised coronal sections of a rat brain stereotaxic atlas (Paxinos and Watson, 1998).

**Experiment 2: Effects of venlafaxine and ketamine on learning and induction of an affective bias**

#### CeN lesions: Rats were anesthetised with isoflurane/O2 and secured in a stereotaxic frame. Bilateral excitotoxic lesions were induced with ibotenic acid (10 ug/ul in 0.1M PBS) injected into the central nucleus of the amygdala from a 2ul Hamilton syringe at a rate of 0.1 µl/min according to the stereotaxic coordinates in table 1. The injector was left in place for 2 min before and 3 min after each injection. The animal was left for 10 minutes after the last injection before recovery from the anaesthetic. After surgery, the animals were housed individually overnight then allowed 18-21 days recovery in normal paired housing conditions. Based on the histology, five animals were excluded from the analysis due to unilaterial lesions or lesions which extended beyond the CeA.

| Number of injections per side | Co-ordinates of each injection site | | |  | Volume per site (µl) | Time between infusion and removal of injector (min) |
| --- | --- | --- | --- | --- | --- | --- |
|  | AP | ML | DV |  |  |  |
| 2 | -1.9-2.6 | ±3.9±4.2 | -8.6-8.3 |  | 0.1ul0.1ul | 3 |
|  |  |  |  |  |  |  |

#### Table 2: Coordinates used for the CeA lesions.

#### Immunohistochemistry: The animals were anesthetised with a lethal dose of sodium pentobarbitone (0.5ml Euthatal, 200 mg/ml, Genus Express, UK) and perfused via the left ventricle with 0.01M PBS followed by 4% paraformaldehyde. The brains were removed and postfixed for 24h in paraformaldehyde. The brains were transferred to 30% sucrose in 0.1 M PBS and left for 2 days before coronal sections were cut at 40μm on a freezing microtome. For NeuN immunostaining sections were incubated with 3% H_2_O_2_ for 10 min to block endogenous peroxidase activity. After washing in PBS containing 0.2% Triton-X100 (PBS-T) (3×5 min), the sections were treated for 30 min with blocking solution (3% normal horse serum, 2% bovine serum albumin in PBS-T [Sigma-Aldrich]). After washing in PBS-T, they were incubated overnight at room temperature with monoclonal mouse anti-NeuN antibody (1:1000, Chemicon). After washing in PBS-T, sections were incubated for 90 min with horse anti-mouse biotinylated antibody (1:1000, Vector). After washhing in PBS-T, sections were incubated for 90 min with extravadin peroxidise (1:1000, Sigma-Aldrich) in PBS and following washing in PBS, colour development was achieved by incubating with 3,3′ diaminobenzidine tetrahydrochloride (DAB; Vector DAB kit SK4100) for 2 min. Sections were then mounted onto slides and left overnight before being dehydrated through graded alcohols, cleared in xylene, and coverslipped in DPX mountant (Sigma-Aldrich). The slides were examined using a Leitz light microscope (Leica, Milton Keynes, UK) to assess the extent of excitotoxin-induced damage. Areas of neuronal loss were mapped onto standardised coronal sections of the rat brain stereotaxic atlas (Paxinos and Watson, 1998) by an experimenter blind to treatment.
