# Supplementary material for: Distinct Neuropsychological Mechanisms May Explain Delayed- Versus Rapid-Onset Antidepressant Efficacy
Source: Neuropsychopharmacology. 2015 Mar 25;40(9):2165–74. doi: 10.1038/npp.2015.59 (PMC4487826; doi:10.1038/npp.2015.59)
Supplement: Supplementary Table S1 [file npp201559x2.docx]

**Table S1 – Protocol overview**

**Protocol 1: Standard procedure for testing treatment-induced affective bias versus control**

Each animal receives drug treatment or vehicle counterbalanced over the four associative learning/pairing sessions. Substrate and day are also counter-balanced resulting in four different groups.

**Protocol 2: Modified procedure for testing additive effects of treatment-induced affective bias versus control over multiple weeks**

Each animal receives drug treatment or vehicle counterbalanced over the two associative learning/pairing sessions. Substrate and day are also counter-balanced resulting in four different groups. The protocol is repeated each week using the same substrates and treatments.
