# Supplementary material for: Distinct Neuropsychological Mechanisms May Explain Delayed- Versus Rapid-Onset Antidepressant Efficacy
Source: Neuropsychopharmacology. 2015 Mar 25;40(9):2165–74. doi: 10.1038/npp.2015.59 (PMC4487826; doi:10.1038/npp.2015.59)
Supplement: Supplementary Table S2 [file npp201559x3.docx]

**Table S2 - Experiment 1**

**Study A – Effect of venlafaxine (3mg/kg) treatment on FG7142-induced negative bias**

**Wk 1**

**Wk 2**

**Rat ID**

**Pairing 1**

**Pairing 2**

**Pairing 3**

**Pairing 4**

**Preference test**

**Pairing 1**

**Pairing 2**

**Pairing 3**

**Pairing 4**

**Preference test**

Rat_1

Veh, Sub A

FG, Sub B

Veh, Sub A

FG, Sub B

Veh

Veh, Sub A

FG, Sub B

Veh, Sub A

FG, Sub B

Vfx

Rat_2

Veh, Sub B

FG, Sub A

Veh, Sub B

FG, Sub A

Veh

Veh, Sub B

FG, Sub A

Veh, Sub B

FG, Sub A

Vfx

Rat_3

FG, Sub A

Veh, Sub B

FG, Sub A

Veh, Sub B

Vfx

FG, Sub A

Veh, Sub B

FG, Sub A

Veh, Sub B

Veh

Rat_4

FG, Sub B

Veh, Sub A

FG, Sub B

Veh, Sub A

Vfx

FG, Sub B

Veh, Sub A

FG, Sub B

Veh, Sub A

Veh

Rat_5

Veh, Sub A

FG, Sub B

Veh, Sub A

FG, Sub B

Veh

Veh, Sub A

FG, Sub B

Veh, Sub A

FG, Sub B

Vfx

Rat_6

Veh, Sub B

FG, Sub A

Veh, Sub B

FG, Sub A

Veh

Veh, Sub B

FG, Sub A

Veh, Sub B

FG, Sub A

Vfx

Rat_7

FG, Sub A

Veh, Sub B

FG, Sub A

Veh, Sub B

Vfx

FG, Sub A

Veh, Sub B

FG, Sub A

Veh, Sub B

Veh

Rat_8

FG, Sub B

Veh, Sub A

FG, Sub B

Veh, Sub A

Vfx

FG, Sub B

Veh, Sub A

FG, Sub B

Veh, Sub A

Veh

Rat_9

Veh, Sub A

FG, Sub B

Veh, Sub A

FG, Sub B

Veh

Veh, Sub A

FG, Sub B

Veh, Sub A

FG, Sub B

Vfx

Rat_10

Veh, Sub B

FG, Sub A

Veh, Sub B

FG, Sub A

Veh

Veh, Sub B

FG, Sub A

Veh, Sub B

FG, Sub A

Vfx

Rat_11

FG, Sub A

Veh, Sub B

FG, Sub A

Veh, Sub B

Vfx

FG, Sub A

Veh, Sub B

FG, Sub A

Veh, Sub B

Veh

Rat_12

FG, Sub B

Veh, Sub A

FG, Sub B

Veh, Sub A

Vfx

FG, Sub B

Veh, Sub A

FG, Sub B

Veh, Sub A

Veh

Rat_13

Veh, Sub A

FG, Sub B

Veh, Sub A

FG, Sub B

Veh

Veh, Sub A

FG, Sub B

Veh, Sub A

FG, Sub B

Vfx

Rat_14

Veh, Sub B

FG, Sub A

Veh, Sub B

FG, Sub A

Veh

Veh, Sub B

FG, Sub A

Veh, Sub B

FG, Sub A

Vfx

Rat_15

FG, Sub A

Veh, Sub B

FG, Sub A

Veh, Sub B

Vfx

FG, Sub A

Veh, Sub B

FG, Sub A

Veh, Sub B

Veh

Rat_16

FG, Sub B

Veh, Sub A

FG, Sub B

Veh, Sub A

Vfx

FG, Sub B

Veh, Sub A

FG, Sub B

Veh, Sub A

Veh

**Study B – Effect of ketamine treatment on FG7142-induced negative bias**

Procedure repeated for ketamine (1mg/kg) using the same design

**Study C – Effect of ketamine on stress-induced negative bias**

Procedure repeated but with restraint stress and social isolation replacing the FG7142 treatment and control housing replacing vehicle treatment (see also design for expt 4).
