# Supplementary material for: Distinct Neuropsychological Mechanisms May Explain Delayed- Versus Rapid-Onset Antidepressant Efficacy
Source: Neuropsychopharmacology. 2015 Mar 25;40(9):2165–74. doi: 10.1038/npp.2015.59 (PMC4487826; doi:10.1038/npp.2015.59)
Supplement: Supplementary Table S3 [file npp201559x4.docx]

**Table S3 - Experiment 2 – Effects of intra-mPFC infusions of vehicle, ketamine, bupivacaine or muscimol on FG7142-induced negative bias**
