# Supplementary material for: Distinct Neuropsychological Mechanisms May Explain Delayed- Versus Rapid-Onset Antidepressant Efficacy
Source: Neuropsychopharmacology. 2015 Mar 25;40(9):2165–74. doi: 10.1038/npp.2015.59 (PMC4487826; doi:10.1038/npp.2015.59)
Supplement: Supplementary Table S4 [file npp201559x5.docx]

**Table S4 - Experiment 2**

**Study A – Effects of ketamine treatment on new learning**

Treatment was randomised over the three weeks of the study as follows:

**ID**

**Wk 1**

**Wk 2**

**Wk 3**

**Rat_1**

0.0

3.0

1.0

**Rat_2**

0.0

3.0

1.0

**Rat_3**

1.0

0.0

3.0

**Rat_4**

1.0

0.0

3.0

**Rat_5**

3.0

1.0

0.0

**Rat_6**

3.0

1.0

0.0

**Rat_7**

0.0

3.0

1.0

**Rat_8**

0.0

3.0

1.0

**Rat_9**

1.0

0.0

3.0

**Rat_10**

1.0

0.0

3.0

**Rat_11**

3.0

1.0

0.0

**Rat_12**

3.0

1.0

0.0

**Rat_13**

0.0

3.0

1.0

**Rat_14**

0.0

3.0

1.0

**Rat_15**

1.0

0.0

3.0

**Rat_16**

1.0

0.0

3.0

**Ketamine dose (mg/kg)**
