# Supplementary material for: Distinct Neuropsychological Mechanisms May Explain Delayed- Versus Rapid-Onset Antidepressant Efficacy
Source: Neuropsychopharmacology. 2015 Mar 25;40(9):2165–74. doi: 10.1038/npp.2015.59 (PMC4487826; doi:10.1038/npp.2015.59)
Supplement: Supplementary Table S5 [file npp201559x6.docx]

**Table S5 – Experiment 2**

**Study A - Effects of amygdala lesions on antidepressant and pro-depressant-induced affective bias**

Group A = Sham, Group B = Lesion

Three way counter-balanced design using protocol 1 where ‘Treatment’ was:

**Study B - Effects of amygdala lesions on stress-induced negative bias**
