# Supplementary material for: Distinct Neuropsychological Mechanisms May Explain Delayed- Versus Rapid-Onset Antidepressant Efficacy
Source: Neuropsychopharmacology. 2015 Mar 25;40(9):2165–74. doi: 10.1038/npp.2015.59 (PMC4487826; doi:10.1038/npp.2015.59)
Supplement: Supplementary Table S6 [file npp201559x7.docx]

**Table S6 – Pairing session data following treatment with ketamine**

|  | **Dose**  **(mg/kg)** | **Choice Latency (sec)** | |  | **Trials to criterion** | |
| --- | --- | --- | --- | --- | --- | --- |
|  |  | **Treatment** | **Vehicle** |  | **Treatment** | **Vehicle** |
| **Ketamine** | 0.0 | 2.1±0.1 | 2.1±0.1 |  | 7.6±0.3 | 6.7±0.5 |
|  | 1.0 | 2.1±0.1 | 2.1±0.0 |  | 6.9±0.2 | 7.5±0.3 |
|  | 3.0 | **2.2±0.1**** | 2.0±0.1 |  | 7.3±0.3 | 7.7±0.3 |

Results for response latency and trials to criteria during pairing sessions following treatment with ketamine. Animals were treated with either drug or vehicle 60min prior to each pairing session using a fully counter-balanced study design. Data represent the mean value obtained from the two pairing sessions performed under each condition. Ketamine tended to slow the animals response latency at the highest dose. No significant effects on choice latency or trials to criterion were observed following treatment with FG7142. Data presented as mean ± s.e.m., n=16 animals per group. ** p<0.01, paired t-test.
