# Supplementary material for: Distinct Neuropsychological Mechanisms May Explain Delayed- Versus Rapid-Onset Antidepressant Efficacy
Source: Neuropsychopharmacology. 2015 Mar 25;40(9):2165–74. doi: 10.1038/npp.2015.59 (PMC4487826; doi:10.1038/npp.2015.59)
Supplement: Supplementary Table S7 [file npp201559x8.docx]

**Table S7 – Pairing session data following treatment with venlafaxine, FG7142, or control in CeA lesioned and sham animals.**

| **Study** | **Group** | **Choice Latency (sec)** | |  | **Trials to criterion** | |
| --- | --- | --- | --- | --- | --- | --- |
|  |  | **Treatment** | **Control** |  | **Treatment** | **Control** |
| **Control** | Sham | 3.7±0.5 | 4.2±0.4 |  | 6.7±0.2 | 6.9±0.2 |
| (*veh vs. veh*) | Lesion | 4.2±0.6 | 4.2±0.4 |  | 7.3±0.3 | 6.6±0.2 |
| **Venlafaxine** | Sham | 4.2±0.2 | 4.0±0.4 |  | 7.3±0.1 | 7.0±0.2 |
|  | Lesion | 4.6±0.4 | 4.4±0.5 |  | 6.7±0.2 | 6.7±0.3 |
| **FG 7142** | Sham | 4.2±0.3 | 4.0±0.3 |  | 6.9±0.3 | 7.2±0.3 |
|  | Lesion | 4.5±0.3 | 3.9±0.3 |  | 6.7±0.2 | 6.8±0.3 |
| **Psychosocial** | Sham | 3.0±0.2 | 3.1±0.2 |  | 6.6±0.2 | 7.0±0.3 |
| **stress** | Lesion | 2.9±0.2 | 2.9±0.2 |  | 6.6±0.2 | 6.8±0.2 |

Results for response latency and trials to criteria during pairing sessions following treatment with vehicle, venlafaxine or FG7142 in sham on amygdala lesioned animals. Animals were treated with either drug or vehicle 30min prior to each pairing session using a fully counter-balanced study design. Data represent the mean value obtained from the two pairing sessions performed under each condition. Data presented as mean ± s.e.m., n=11 lesioned, n=16 sham.
